# Simulation of the emergence of cell-like morphologies with evolutionary potential based on virtual molecular interactions

**DOI:** 10.1038/s41598-024-52475-9

**Published:** 2024-01-24

**Authors:** Takeshi Ishida

**Affiliations:** Department of Ocean Mechanical Engineering, National Fisheries University, 2-7-1, Nagata-Honmachi, Shimonoseki, Yamaguchi 759-6595 Japan

**Keywords:** Computational biophysics, Computational models

## Abstract

This study explored the emergence of life using a simulation model approach. The “multiset chemical lattice model” allows the placement of virtual molecules of multiple types in each lattice cell in a two-dimensional space. This model was capable of describing a wide variety of states and interactions, such as the diffusion, chemical reaction, and polymerization of virtual molecules, in a limited number of lattice cell spaces. Moreover, this model was capable of describing a wide variety of states and interactions, even in the limited lattice cell space of 100 × 100 cells. In this study, I assumed 18 types of virtual molecules, i.e., 18 virtual numbers that do not correspond to real molecules with chemical reactions represented by transformation of the numbers that occur with a specified reaction rate probability. Furthermore, it considered the energy metabolism and energy resources in the environment, and was able to reproduce “evolution,” in which a certain cell-like shape that adapted to the environment survived under conditions of decreasing amounts of energy resources in the environment. This enabled the simulation of the emergence of cell-like shapes with the four minimum cellular requirements, i.e., boundary, metabolism, replication, and evolution, based solely on the interaction of virtual molecules.

## Introduction

The clarification of the process of emergence of the first life remains one of the great challenges of science. With the goal of examining this process, research on the origin of life has long been conducted, and many hypotheses, such as the RNA world hypothesis^1^, have been published, for example^2,3^.

When studying the origin of life, it is necessary to consider the conditions that are necessary to determine the emergence of life. The definition of life as a premise is a source of debate and needs to be clarified. In this study, the four conditions that are generally accepted were considered, i.e., structures that are (1) bounded, (2) replicating, (3) metabolizing energy, and (4) able to inherit information and to evolve. However, none one of these conditions alone can be called life; rather, to reach the level of life, these functions must be fulfilled simultaneously.

Various approaches to the study of the process underlying the emergence of the first cell satisfying these four conditions are being undertaken, such as studies examining various chemical evolutions^4,5^, experimental synthesis of minimal cells (protocells)^6,7^, computer-assisted artificial life studies^8,9^, and theoretical studies of complex systems^10^.

Each of these research approaches also provided a poor explanation of how the four conditions for life were simultaneously established. Even assuming the creation of the first RNA, little is known about the process through which it acquired boundaries, metabolism, and replication functions. Therefore, it is not always clear how a chemical process that simultaneously satisfies all four conditions emerged after the materials for life were available.

Thus, the mechanism via which a persistent and evolving cell-like form can emerge under conditions of molecular interaction alone remains a major mystery. Furthermore, its elucidation will not only increase the possibility of determining the origin of life and of synthesizing the smallest cells (protocells), but also be applicable to the emergent construction and mass production of various molecular machines at the micro and nano level.

This study explored the emergence of life through a simulation model approach. Several similar research approaches have been reported. In the RNA world hypothesis, which is currently the leading one, provides examples of computer models that can examine the processes leading to the RNA world. Szilágyi et al.^11^ investigated the robust co-evolution of catalytically active, metabolically cooperating prebiotic RNA replicators using an RNA world model of the origin of life based on physically and chemically plausible first principles. Yin et al.^12^ and others have similarly constructed computer models of the emergence of RNA worlds.

Other computational approaches were as follows: (1) probabilistic models or discrete models, such as cellular automata or artificial chemistry; and (2) calculation of protocells based on molecular dynamics models. Lenia is a recent example of artificial life^13^. This is a model based on Life Games that spins out life-like movements, but does not necessarily simulate the emergence of life’s four conditions. Schmickl^14^ generated a simple motion law for moving and interacting self-propelled particles, leading to a self-structuring, self-reproducing, and self-sustaining life-like system. The patterns emerging within this system resembled the patterns found in living organisms.

Regarding research on the process of first life based on artificial chemistry, Kruszewski et al.^15^ validated self-reproducing metabolisms with a minimalistic artificial chemistry based on a Turing-complete rewriting system called combinatory logic. Fellermann^16^ performed a dissipative particle dynamics simulation that combined self-assembly processes with chemical reaction networks. Moreover, Hutton^17^ constructed a replication model based on the interaction of two-dimensional particle swarms.

Molecular dynamics (MD) is a modeling method for calculating molecule behavior that requires high computation to calculate the behavior of a small number of molecules, as it solves each individual molecule mechanically. This method can calculate only a limited time within a limited space, thus rendering it difficult to compute a single whole living cell. Therefore, reducing the number of degrees of freedom is necessary to decrease the computational complexity. There are many reports on coarse-grained models of MD, including computational examples of the self-organizing structure of cell membranes^8,18^ and of cell membrane division^19^ and simulations of nanoscale mechanisms for the budding and fission of nanovesicles^20^. In addition, stochastic–deterministic simulations over a cell cycle using a whole-cell fully dynamical kinetic model have been reported^9^. These studies are simulations of each protocell construction process; however, they do not refer to the emergence of protocells from molecules or the acquisition of the four conditions of life.

**Figure 1:**
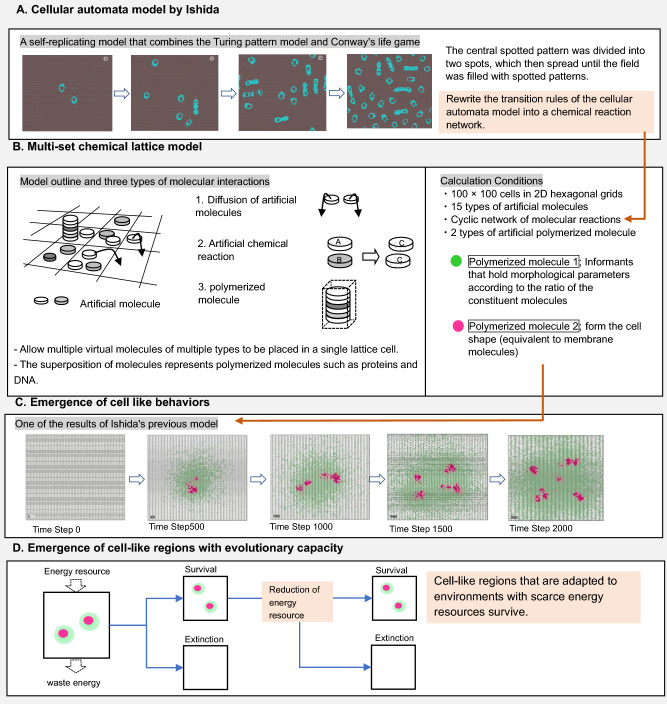
Schematic overview of the “Multiset chemical lattice model”. (**A**) Ishida^21^ improved the cellular automata model and found various shapes, such as self-replication. (**B**) This model allows the placement of multiple virtual molecules of multiple types in a single cell on a two-dimensional lattice space. The application of discrete stochastic transitions to each molecule allows the description of a wide variety of states and interactions in a limited number of cells, such as the migration (diffusion), transformation (chemical reaction), and linkage (polymerization) of virtual molecules. (**C**) Distribution of the two types of polymerized molecules. In the initial stage of the time step, an area of accumulation of red polymerized molecules was formed in the center of the lattice field, followed by the continuous disruption of this region of polymerized molecules. Concomitantly, it was confirmed that the information of the w value was retained in the green polymerized molecules and transmitted to the space. (**D**) The construction of an extended model that explicitly incorporates energy metabolism reproduced the phenomenon of evolution in which a certain cell-like shape that adapted to the environment survives under conditions of decreasing amounts of energy resources in the environment.

The author, Ishida, has been studying the physicochemical processes that simultaneously emerge under the four conditions of life from the aspect of discrete mathematical modeling. Ishida^21^ improved the Cellular automata (CA) model, is discrete both in space and time, and the state of the focal cell is determined by the states of the adjacent cells and transition rules. Ishida’s CA model is an external sum rule type CA in which the state of a lattice is determined by the sum of the states of neighboring lattices, and found that various shapes, such as self-replication (shown in Fig. 1A), growth, and branching, can be elicited and controlled using a few parameters. Furthermore, Ishida^22^ considered a model that allows the placement of virtual molecules of multiple types in each cell on a two-dimensional space, as shown in Fig. 1B. This model, which is termed the “Multiset chemical lattice model,” is capable of describing a wide variety of states and interactions in a limited number of lattice cell spaces, such as the migration (diffusion), transformation (chemical reaction), and linkage (polymerization) of virtual molecules. This model is capable of describing a wide variety of states and interactions even in the limited lattice cell space of 100 × 100 cells.

In Ishida’s model^22^, although 15 types of artificial molecules are assumed, the number of chemical reaction combinations among the 15 types of molecules is enormous, and it is difficult to identify the reactions among these combinations that trigger life emergence phenomena. Ishida^22^ converted the algorithm used for generating Turing patterns reported in the author’s articles^21,23^ into procedures for the diffusion and reaction of the 15 different virtual molecules and the polymerization of virtual molecules. Using only the processes of molecular diffusion, reaction, and polymerization, as shown in Fig. 1C, this model was able to realize a process in which metabolism begins, boundaries are created, and these boundaries replicate while retaining the information necessary for the maintenance of form (equivalent to genetic information).

However, although Ishida’s model^22^ calculated the phenomena of the simultaneous emergence of “boundary formation,” “replication,” and “metabolism,” it was unable to specify whether the cell-like region retains evolutionary capacity. In this report, using this lattice-type multiset chemical model, an extended model was constructed that explicitly incorporated energy metabolism. And as shown in Fig. 1D, the model was able to reproduce the phenomenon (i.e., “evolution”) in which a certain cell-like shape that adapted to the environment survived under conditions of decreasing amounts of energy resources in the environment.

This enabled the emergence of a cell-like morphology with the four minimum cellular requirements (boundary, metabolism, replication, and evolution) based solely on the interaction of virtual molecules. Although this study neither provided a new hypothesis of life emergence nor proved a conventional hypothesis, it was an important step in the clarification of the cellular emergence process.

In particular, the model assumed that the formation and decomposition of polymers consisting of 100 molecules linked together can be easily realized. However, this cannot be achieved in a natural reaction environment unless a catalyst is already present in biochemical reactions, and difficulties remain in explaining the first life-emerging processes.

However, it proposed a concrete and simple mathematical model to address the issue that, to date, has only been vaguely expressed as “the conditions for life have been established as a result of a long process of trial and error.” If it is possible to create a reaction system similar to the reaction process described in this article through experimental approaches in the future, it will be possible to create cell-like capsules and to mass produce microcapsules.

## Results

### Simulation of cell-like shapes under conditions of abundant supply of energy resources

I applied the multiset chemical lattice model, adding energy metabolism reactions to Ishida’s model^22^, on a 100 × 100 hexagonal lattice space. In this study, I summarized the results related to the differences in the energy resource supply. In addition, I confirmed whether genes that favor energy production would survive in environments with scarce energy resources.

**Figure 2:**
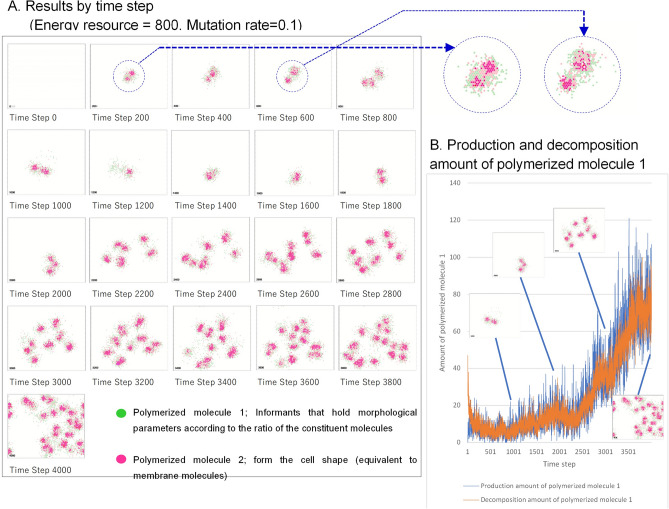
Time series of a case where the amount of energy resources E input into the computational space was 800. (**A**) Polymerized molecule 2, which is responsible for cell morphology, is shown in red. Under conditions of abundant energy resources, this cell-like shape emerged and divided. (**B**) Amount of polymerized molecule 1 (green area in the figure), which plays the role of an informant, that was generated and degraded. As the cell-like regions divided and increased in number within the computational domain, the amount of polymerized molecule 1 (informant) that was produced and degraded also increased.

The results of the time series of the case in which the amount of energy resources E input into the computational space was 800 are shown in Fig. 2A. In the figure, polymerized molecule 2, which was responsible for cell morphology, is shown in red. Under conditions of abundant energy resources, this cell-like shape emerged and divided, reproducing the same phenomenon as that reported for Ishida’s previous model.

Even if the initial conditions and parameter values were the same, the pattern of the cell-like morphology changed with each calculation because excess molecules that cannot be divided into the six orientations in the molecular diffusion process are assigned to the orientations via random numbers, and because, for some parts, the presence or absence of molecular conversion is determined probabilistically based on reaction probabilities.

Figure 2B reports the amount of polymerized molecule 1 (green area in the figure), which played the role of an informant, that was generated and degraded. As the cell-like regions divided and increased in number within the computational domain, the amount of polymerized molecule 1 (informant) that was produced and degraded also increased. Thus, informants were repeatedly generated and decomposed, and, by introducing the action of selection through mutation, it was possible to change the information held by informants.

### Results of the energy resource supply

**Figure 3:**
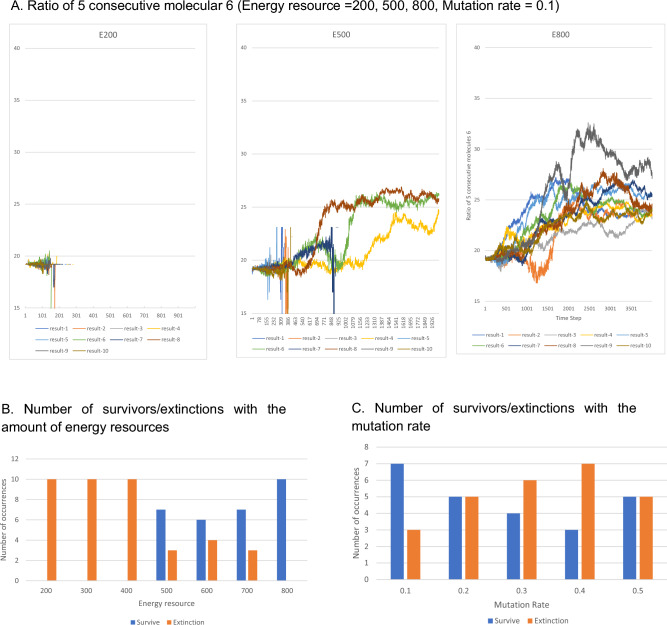
Results of the calculation based on different levels of energy resources and mutation rates. (**A**) Results of 10 calculations performed by varying the amount of energy resource E (the amount of new energy supply when the number of molecule 20 was zero in each lattice cell). The vertical axis in A indicates the evolution of the ratio of the number of five consecutive molecules 6 in the polymerized molecule. (**B**) Results of 10 trials for each energy resource E, showing the number of survival and disappearance events. When the resource supply E exceeded 500, the number of cases that survived was increased. A greater supply of energy resource E implied an increase in the survival of the cell-like regions. (**C**) Results obtained for a varying mutation rate. This figure shows the number of survival and extinction events for each of the 10 trials performed with different mutation rates in the case of energy resource E = 500.

The results of 10 calculations for varying amounts of the energy resource E (the amount of new energy supply when the number of molecule 20 was zero in each lattice cell) are shown in Fig. 3A. The vertical axis in Fig. 3A indicates the evolution of the ratio of the number of five consecutive molecules 6 in the polymerized molecule. As mentioned above, when molecules 6 and 7 in polymerized molecule 1 were randomly exchanged, five consecutive molecules 6 had an average occurrence rate of 19% when the composition ratio of molecule 6 was 0.68. All of the surviving computational cases in the 10 trials had an occurrence rate of 19% or higher, indicating that cell-like regions with a high occurrence rate of polymerized molecule 1 survived in the computational lattice space. Cell-like regions with informants that had a larger ratio of five consecutive molecules 6 were more likely to survive by producing more energy molecules 25. When the energy resource supply E was lower, the amount of energy resource input into the lattice space was reduced and the number of cases in which the cell shape disappeared in a short time step increased.

Figure 3B reports the results of 10 trials for each energy resource E, showing the number of survival and disappearance events. When the resource supply E exceeded 500, the number of surviving cases was increased. Therefore, a greater supply of energy resource E implied an increased tendency to survive of the cell-like regions.

### Results of the mutation rates of polymerized molecule 1

In the standard case, the mutation of polymerized molecule 1 was set to 0.1. Figure 3C depicts the results obtained when the mutation rate varied. This figure indicates the number of survival and extinction events for each of the 10 trials with different mutation rates in the case of energy resource E = 500. The results showed that higher mutation rates did not increase the number of surviving cell-like regions, probably because a high mutation rate increases the frequency of replacement of molecules 6 and 7, thus hampering the maintenance of five consecutive portions of molecule 6 for a long period; i.e., the survival ratio cannot be maintained at a high level.

### Results of the calculation performed in an environment of decreasing energy resource supply

**Figure 4:**
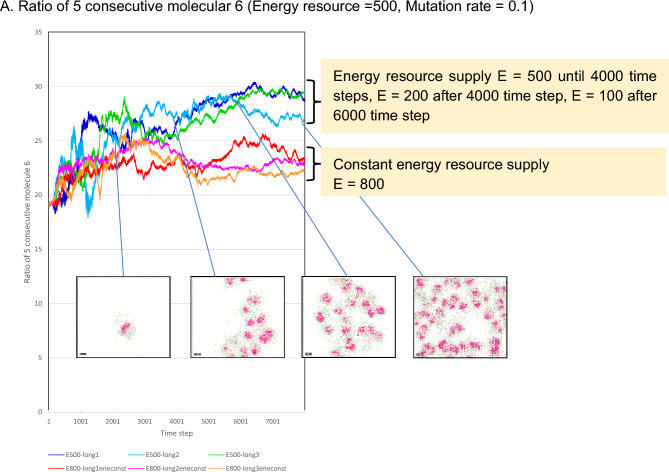
Results of the calculations performed in an environment of a decreasing energy resource supply. (**A**) Results obtained by reducing the amount of energy resources supplied to the grid space over time. As in Fig. 3A, the ratio of the number of occurrences of five consecutive molecules 6 in polymerized molecule 1 is shown. In the figure, the results of the calculations are shown for three cases, one in which the amount of energy resources E was kept constant at 800, and another three cases in which the amount of energy resources was maintained at 800 until step 4000, reduced to E = 200 after step 4000, and reduced to E = 100 after step 6000.

Figure 4 reports the results obtained by reducing the amount of energy resources supplied to the lattice space over time. As in Fig. 3A, the ratio of the number of occurrences of five consecutive molecules 6 in polymerized molecule 1 is shown. In the figure, the results of the calculations are indicated for two calculation patterns; first three cases are one in which the amount of energy resources E was kept constant at 800, and another three cases in which the amount of energy resources was maintained at 800 until step 4000, reduced to E = 200 after step 4000, and reduced to E = 100 after step 6000.

This result suggests that the cell-like regions will survive even when the amount of energy resources supplied to the lattice space is reduced. Figure 3B indicates that the environment created at E = 200 is difficult for cell-like regions to survive, but cell-like regions survived even when the amount of energy resources was 200 or 100 after the 4000th step. This finding suggests that regions that acquired the high number of consecutive sections of five molecules 6 were able to survive.

## Discussion

In this model, 18 artificial molecules were assumed, and the unique residual rate of each molecule was set, as well as the reaction probability of intermolecular reactions and the molecular ratio of molecule 6 to molecule 7 of polymerized molecule 1 (representing the parameter of morphology). Molecules were placed in each cell as initial values, and the local numerical manipulation of the diffusion alone, reaction, polymerization, and decomposition of each type of molecule allowed us to simulate the emergence and replication of cell-like shapes in which polymerized molecule 2 aggregated. A model was constructed in which the information on the morphological parameter was retained in the form of the molecular configuration ratio of polymerized molecule 1, and this configuration ratio was inherited in the process of the emergence and disappearance of self-replicating regions.

Furthermore, a mutation process was introduced in which the composition ratio of molecules 6 and 7 of polymerized molecule 1 was not changed, whereas the order of the molecules was randomly changed and a setting in which the amount of energy (molecule 25) production changed according to the number of five consecutive molecules 6 in polymerized molecule 1 was introduced.

If the ratio of molecule 6 to molecule 7 in polymerized molecule 1 was maintained at 68:32 and molecules 6 and 7 were randomly exchanged, the average ratio of five consecutive molecules 6 was 19%. However, when looking at the calculation cases in which the generation, division, and disappearance of cell-like shapes continued, the ratio of five consecutive cases became higher than 19% in all cases. This means that if, during the mutation process, the reaction network that maintains the cell shape fails to maintain the shape because of the lack of energy molecules (molecule 25) when the ratio of five consecutive molecules 6 is not high, the cell-shaped region will disappear. Conversely, a phenomenon is expected to be observed in which the cell-like shape that retains the polymerized molecule 1 with a high ratio of five contiguous molecules 6 survives.

When the amount of energy resources (molecule 20) input into the computational lattice space varied, more abundant energy resources implied an increase in the survival of the cell-like shapes. Furthermore, when the amount of the energy resource input was reduced after a certain time step, the cell-like region that retained the polymerized molecule 1 with a high ratio of five consecutive molecules 6 survived. Because this region was able to maintain high energy production, it is thought that the cell shape can survive and adapt to environments with low energy resources (which would not normally be conducive to survival). The model suggests that, even in an environment with limited energy resources, individuals with informants that can generate a great amount of energy will survive; therefore, it is thought that a basic process of evolution in response to the environment was created.

The surviving cell-like regions were found to have a greater ratio of five conservative molecules 6 than the random average. This corresponds to spontaneous selection for genetic information (survival of individuals that happen to be in favorable conditions). This suggests a primitive evolution, in which cells with more favorable informants than the average survive.

In the previous Ishida^23^ model, only three types of cell shape emergence, replication, and metabolism were realized; in contrast, the present model may have shown that the emerged cellular shape also possessed “evolutionary potential.”

Based on the Ishida^23^ model, a model was constructed that explicitly incorporated reactions of energy metabolism by adding three new molecules in addition to the 15 original molecules. This model was able to produce cell-like shapes with the ability to evolve to an energy resource environment. Based on this model, a cell-like shape with the four conditions of a cell, i.e., boundary, metabolism, replication, and evolution, could be emergently created by assuming several chemical reactions and molecular polymerization.

Although this model remains a simple model at present, as the number of molecule types increase and the degree of freedom of the reaction increases, and under energy supply that is sufficient to sustain the reaction, new reaction paths and multiple reaction loops may be formed. This could potentially reproduce the process of evolution to more complex cells. The potential application of this mechanism to actual chemical reactions may provide a basis for considering the synthesis of minimal cells (protocells) or the self-organized synthesis of nanomachines.

Conversely, the weakness of this model lies in that it assumed that the formation and decomposition of 100 linked polymerized molecules can be easily realized. In fact, this cannot be easily achieved in a natural reaction environment unless a catalyst or other means are already present. Several difficulties remain regarding the use of this model to explain the emergent process in which the first life simultaneously maintained all four conditions in the absence of a catalyst.

To solve this issue, as the next stage of research, it is necessary to construct a computational model that includes the emergence process of long polymerized molecules with catalytic functions, starting from single molecules and light polymerized molecules with a few molecules (which can polymerize and degrade naturally via the action of minerals or other substances without protein catalysts), and simulate life emergence including “catalyst emergence.”

## Model

### Overview of the multiset chemical lattice model

#### Model configuration

In this study, an extended two-dimensional lattice-type multiset chemical model, as reported by Ishida^22^, was constructed. This Ishida model applied a multiset chemical model in lattice space. The computational representation of chemical reactions has been modeled as a multiset chemical model, which is also known as an “artificial chemical model”^24^. Multiset refers to the concept of a mathematical class plus multiplicity. Using the multiset concept, it was possible to construct a model that took into account the number of molecules as well as the type of molecules. For example, considering molecules **a** and **b**, a multiset representation of the state with three molecules **a** and two molecules **b** would be {**a**, **a**, **a**, **a**, **b**, **b**}. The transformation of molecule **a** and molecule **b** into molecule **c** through chemical reactions can be expressed by the following multiset rewriting rule, {**a**, **a**, **a**, **b**, **b**} → {**a**, **c**, **c**}.

The rewriting rule can also be set to occur with a certain reaction probability. Once the rewriting rule and the reaction probability of the chemical reaction were determined, this modeling allowed the number of molecules to be rewritten.

In each lattice cell, the number of molecules was recorded for each molecular species. The following is the modeling method of the diffusion, reaction, and polymerization processes of each molecule in the lattice cell.

**Figure 5:**
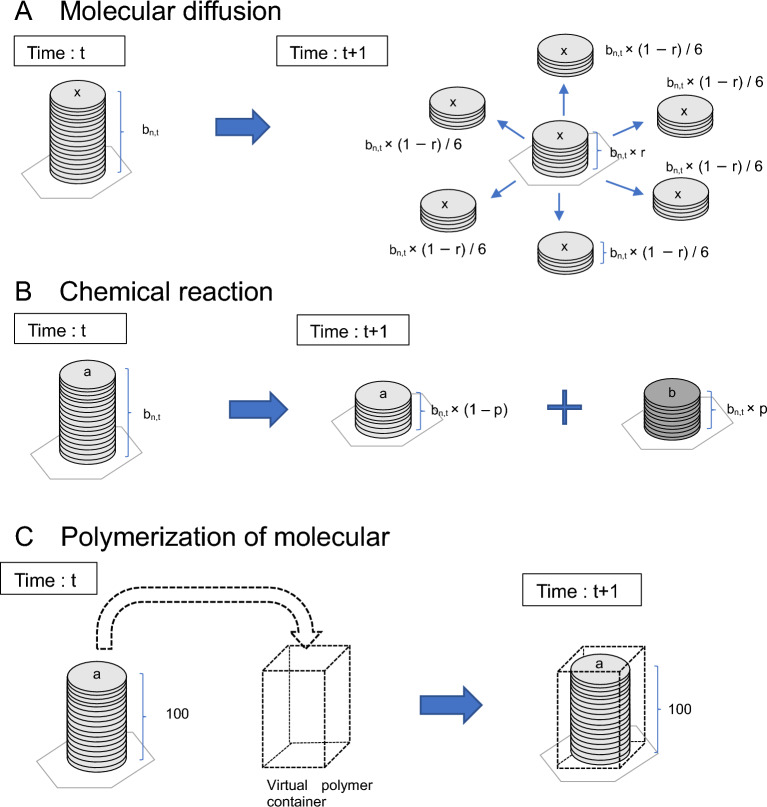
Schematic representation of the diffusion and chemical reaction of molecules, and virtual box model representing the state of the polymers. (**A**) Molecular diffusion can be represented by the exchange of molecular numbers between adjacent cells. In this model, the diffusion of molecules and polymerized molecules in each cell was represented by the following process. The residual rate was defined here as an alternative parameter to the diffusion coefficient. The residual ratio r, as the parameter of each molecular type, was the fraction of unmoved molecules in each cell. The parameter r, as a molecule-specific attribute value, was fixed for all cells and all time steps. The calculations (as shown in the figure) were performed, where b_n,t_ is the number of molecules in cell n at time t: (1) a proportion of the molecules, b_n,t_ × (1 − r) × 1/6, diffused toward the six adjacent cells evenly; (2) the residual molecules, b_n,t_ × r, remained in the original cell. If the number of molecules was not an integer multiple of 6, the remainder of the molecules were distributed between adjacent cells with equal probability. (**B**) Chemical reactions were represented by rewriting the number of molecular types based on reaction probabilities. The figure provides one example of the case of a reaction from molecule a to molecule b at reaction rate p. (**C**) A virtual box filled with virtual molecules collectively was assumed. The empty box in the lattice space was placed to represent the polymerization of molecules when the box was filled with molecules. An empty box, after the removal of the molecules from it, represented a state in which the polymerized molecules were broken down into small molecules.

As shown in Fig. 5A, molecular diffusion can be represented by the exchange of molecular numbers between adjacent cells. In this model, the diffusion of molecules and polymerized molecules in each cell was represented by the process depicted in Fig. 5A. The residual rate ***r*** was defined here as an alternative parameter to the diffusion coefficient.

The residual ratio ***r***, as the parameter of each molecular type, is the fraction of unmoved molecules in each cell. The parameter ***r*** is a molecule-specific attribute value that was fixed for all cells and all time steps. As shown in Fig. 5A, the following calculations were performed (where ***b***_***n,t***_ is the number of molecules in cell ***n*** at time ***t***): (1) a proportion of the molecules, i.e., ***b***_***n,t***_ × (1 − ***r***) × 1/6, diffused toward the six adjacent cells evenly; (2) the residual molecules, ***b***_***n,t***_ × ***r***, remained in the original cell. If the number of molecules was not an integer multiple of 6, the remainder of the molecules were distributed between adjacent cells with equal probability.

Next, as shown in Fig. 5B, chemical reactions were represented by rewriting the number of molecular types based on reaction probabilities according to the multiset rewriting rule. In this model, 15 types of molecules were set up to replace the algorithm of previous studies that applied the Ishida model^21,23^ to molecular reactions, as described below. As this was an artificial chemical model, it did not correspond to real molecules, and molecular species were represented as “molecule 1,” “molecule 2,” etc. The chemical reaction was then assumed to occur at each time step with a certain probability of change in the number of molecules in each cell, as shown in Fig. 5B. For example, in the case of a reaction from molecule **A** to molecule **B**, molecule **A** is converted to molecule **B** with the reaction probability **p**. Assuming that the number of molecules **A** at time t is **A**_**n**_ and the number of molecules **B** is **B**_**n**_, the number of molecules at the next time step is as follows.

$$ \begin{aligned} & {\mathbf{A}}_{{{\mathbf{n}},{\mathbf{t}} + {\mathbf{1}}}} = {\mathbf{A}}_{{{\mathbf{n}},{\mathbf{t}}}} \times ({1}{-}{\mathbf{p}}). \\ & {\mathbf{B}}_{{{\mathbf{n}},{\mathbf{t}} + {\mathbf{1}}}} = {\mathbf{B}}_{{{\mathbf{n}},{\mathbf{t}}}} + {\mathbf{A}}_{{{\mathbf{n}},{\mathbf{t}}}} \times {\mathbf{p}}. \\ \end{aligned} $$

Furthermore, macromolecules such as cell membranes and genes play an essential role in the emergent processes of life; therefore, methods that can be used to represent molecular polymers are necessary. In this model, a virtual box filled with virtual molecules collectively was considered, as shown in Fig. 5C. The empty box in the space was placed in anticipation of the polymerization of molecules when the box was filled with them. When the box was emptied after the removal of the molecules, it represented a state in which the polymerized molecules were broken down into small molecules. Furthermore, the box itself was modeled to be diffuse in the lattice space.

#### Configuration of the molecular reaction network

**Figure 6:**
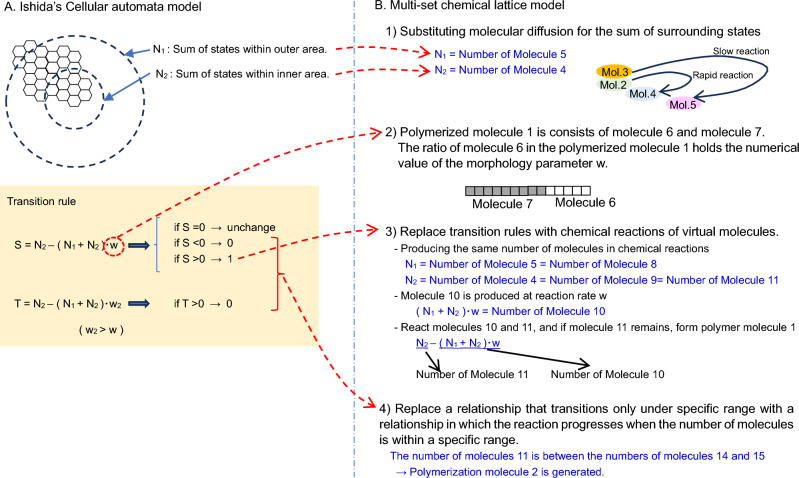
Overview of the transition rule conversions of this CA model to a multiset chemical model. (**A**) Overview of the transition rules of Ishida’s CA model^21^, which combines the Turing pattern model with Conway’s Game of Life. (**B**) In Ishida's multiset model^22^, the sum rule of Ishida’s CA model is replaced by two virtual molecules with molecular changes during diffusion. Furthermore, the operation of the number of states indicated in the transition rule as well as the decision process by inequality are replaced by the molecular changes in chemical reactions and the molecules that become surplus due to molecular reactions respectively.

Based on Ishida’s model^22^, 15 different molecules were set up to replace the algorithm of the model^21,23^, which realized the emergence of a cell-like shape in the cellular automaton model via molecular reactions. Ishida’s model^21^ is a CA model, which combines the Turing pattern model with Conway’s Game of Life. Figure 6A presents an overview of the transition rules for this CA model. This model allows for the creation of diverse patterns (region formation, movement, and replication) in the 2D field. Figure 6B provides an overview of the transition rule conversions of this CA model to the multiset chemical model (see Ishida's model^22^ for details). In Ishida’s multiset model^22^, the process for calculating the sum of states of the CA model is replaced by two virtual molecules changing during diffusion. Furthermore, the operation of the number of states indicated in the transition expression is replaced by the molecular changes in chemical reactions, and the decision process by inequality is replaced by the molecules that become surplus due to molecular reactions. By substituting these processes, I could successfully derive a model that exhibits the same behavior (region formation, moving, and self-replication) as the CA transition rule. The 15 molecules had the following roles.

- Molecule 1: the material to be converted into each molecule (initially, a large number of these molecules were placed in the lattice space).
- Molecules 2 and 3: corresponded to diffusing substances in the Turing pattern model (the difference in the diffusion coefficients was expressed by the difference in their residual rate).
- Molecules 4 and 5: resulting from Molecules 2 and 3 during diffusion, respectively.
- Molecules 6 and 7: materials for “polymerized molecule 1,” which was a polymer of molecules 6 and 7, with the ratio of molecules 6 and 7 representing the morphology parameter in the Ishida model^24^ algorithm; depending on the value of the morphological parameter, the Turing pattern can be controlled from black spots on white, stripe patterns, and white spots on black.
- Molecules 8, 9, 10, and 11: describing the transition equation of the Ishida model^23^ in chemical reactions.
- Molecule 12: the material for “polymerized molecule 2,” which represents the boundary of the cell.
- Molecules 13, 14, and 15: describing the transition equation of the Ishida model^23^ in chemical reactions.

**Figure 7:**
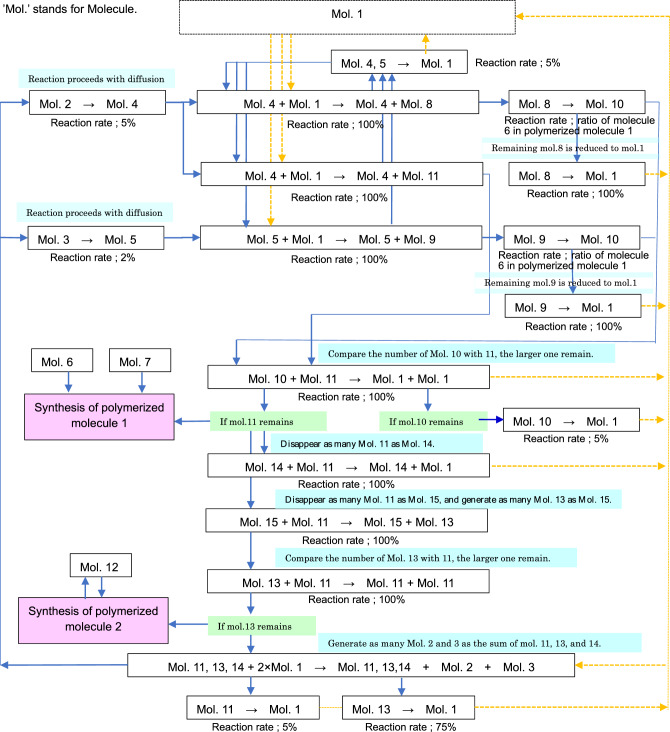
Network map of chemical reactions^22^. Summary of the network of chemical reactions in Ishida’s model^22^. Molecular diffusion and a cyclic network of molecular reactions were constructed to create an algorithm identical to the Ishida model^21^. These reaction networks allow the formation of cell-like shapes and the emergence of self-replication.

Moreover, in Ishida’s model^22^, the whole reaction pathways for each molecule were set up as shown in Fig. 7. By setting up the molecular diffusion and reaction in each lattice as described above, it was possible to realize algorithms similar to those of the Ishida’s CA model^21^ and to generate a variety of forms, including Turing patterns. The reaction like a + b =  > a + c in Fig. 7 indicates that the reaction proceeds under the condition that molecule a is present as a catalyst. The system then set up cyclical reaction paths where molecules decompose at a certain ratio and return to molecule 1; and the overall number of molecules is conserved. Moreover, this chain of reactions is thought to model metabolism concomitantly.

Furthermore, based on the molecular setup designed in this study, new molecules related to energy metabolism and their reactions were added. Specific descriptions are presented in the following section.

### Introduction of energy metabolic reactions

#### Supply of energy resources and their removal from lattice space

The former Ishida model^22^ is built on the assumption of the presence of sufficient energy in the lattice space for all chemical reactions to proceed, and does not explicitly incorporate energy metabolism. In the present study, the model was extended to allow the introduction of molecules that served as energy resources and molecules that were consumed as energy during each chemical reaction.

First, molecule 20 was introduced as an energy resource, with molecule 25 carrying energy to assist the chemical reactions of other molecules and molecule19 being a waste molecule from which energy was taken from the 25 molecule. The resource molecule 20 s were continuously supplied in constant amounts throughout the lattice space, whereas the waste molecule 19 s was excluded from the space. These molecules allowed the explicit inflow and removal of energy into and out of the lattice space.

The reactions of the energy molecules were set up as follows.

$$ {\text{Molecule 2}}0 \to {\text{Molecule 25}}. $$

- *Reaction conditions:* There must be a polymerized molecule 1 with a molecular arrangement that serves as a catalyst.
- *Reaction rate:* It is set that the reaction rate varies according to the amount of a specific catalyst sequence (in which five molecules 6 are connected in succession) in the polymerized molecule 1.

Moreover, in each of the reactions included in the reaction network shown in Fig. 7, molecule 25 was set to be consumed with each reaction as the reaction energy.

$${\text{Molecule 25}} \to {\text{Molecule 19}} + {\text{energy}}.$$

- *Reaction conditions:* Molecule 25 is consumed during the reaction of each molecule according to the number of molecules, and is converted to molecule 19.
- *Reaction rate:* 100%.

Based on the conditions described above, to maintain the cell-like shape in which the polymerized molecules 2 are assembled, molecules 25 must continue to be supplied to the area. For molecule 25 to exist, molecule 20 must be supplied to each lattice cell.

#### Catalytic function set up based on polymerization molecule 1

**Figure 8:**
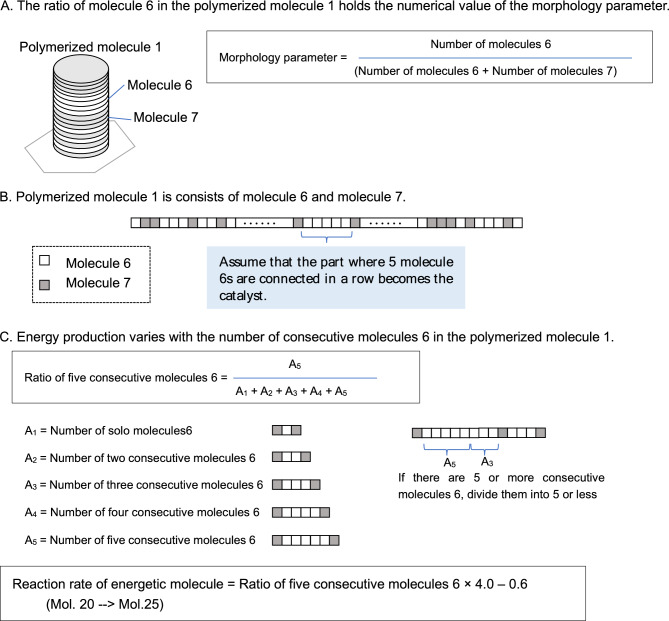
Catalyst modeling. (**A**) Polymerized molecule 1 is composed of molecule 6 and molecule 7 in a specific ratio, with the ratio being a parameter of morphology. (**B**) The ratio of molecule 6 to molecule 7 was used as a parameter, and a specific sequence of molecule 6 was used as a catalyst. Specifically, it was assumed that, if there are five consecutive sections of molecule 6, this part has a catalytic function to produce 25 energy molecules. (**C**) The number of consecutive portions of molecule 6 in polymerization molecule 1 in each lattice was counted, and the reaction probability was set to change according to the number of configurations of five consecutive portions of molecule 6.

In the reaction that converts molecule 20 to molecule 25, polymerized molecule 1 was assumed as the catalyst. As shown in Fig. 8A, polymerized molecule 1 was composed of molecule 6 and molecule 7 in a specific ratio, with the ratio being a parameter of morphology; however, concomitantly, a specific sequence of molecule 6 was assumed to have a catalytic function.

A composition ratio of 0.68 of molecule 6 and molecule 7 (molecule 6/(molecule 6 + molecule 7)) was used in this paper, in reference to the Ishida model^22^ setting. This value was a parameter that allowed the emergence of cell-like shapes and the emergence of a continuously dividing state in the original model, in which the ratio of these molecules alone was used as a parameter for morphogenesis, without considering the order of molecules 6 and 7.

In the present study, the ratio of molecule 6 to molecule 7 was used as a parameter, and a specific sequence of molecule 6 was used as a catalyst. Specifically, as shown in Fig. 8B, it was assumed that, if there were five consecutive sections of molecule 6, this part had a catalytic function to produce energy molecule 25. Such a setting is an analogy of a particular sequence of amino acids performing a particular catalytic function.

Here, as shown in Fig. 8C, the number of consecutive portions of molecule 6 in the polymerization molecule 1 in each lattice was counted, and the reaction probability was set to change according to the number of configurations of five consecutive portions of molecule 6. Although any sequence can be used as the catalytic function, this setting was chosen because it facilitates the simulation of the frequency of five consecutive sections when polymerized molecule 1 was randomly mutated.

The specific reaction probability formulas were set up as follows. If molecules 6 and 7 in polymerized molecule 1 mutated randomly, the composition ratio of five consecutive molecules 6 changed by approximately 0.15–0.3, with the reaction rate changing from 0 to 60%, accordingly.

$${\text{Reaction rate of energy molecule 2}}0 = {\text{Composition ratio of five consecutive molecule 6}} \times {4}.0{-}0.{6}$$

#### Initial configuration of molecule 6 in polymerized molecule 1

**Figure 9:**
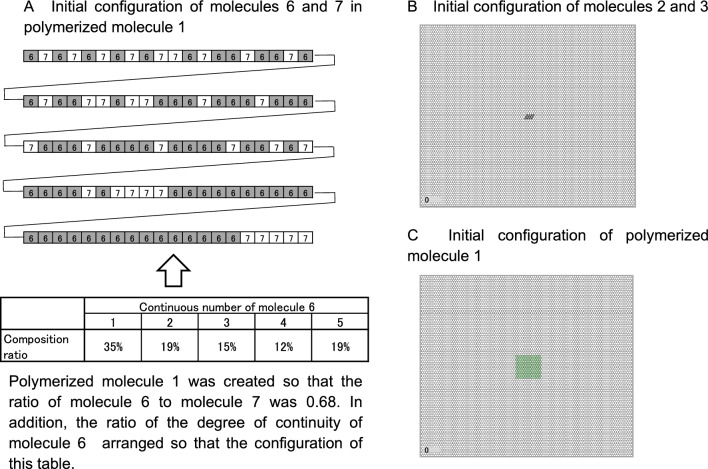
Initial arrangement of molecules in polymerization molecule 1 (catalyst) and initial arrangement of molecules 2 and 3. (**A**) Polymerized molecule 1 was created and arranged so that the ratio of the degree of continuity of molecule 6 was in the configuration shown in the table in (**A**). (**B**) One hundred molecules 2 and 3 were in the central area of the lattice cell field. (**C**) Ten polymerized molecules 1, which were polymerized with ratio molecules 6 and 7 with morphological parameter w, were placed in the central region of the space.

In the initial state of the lattice space, if there are no polymerized molecules 1 at all, no energy molecules 25 will be produced and the reaction network will not proceed; therefore, some polymerized molecules 1 were placed in the lattice space, as an initial value. These polymerized molecules 1 were created and arranged so that the ratio of the degree of continuity of molecule 6 was in the configuration shown in the table in Fig. 9A. This ratio was the average of the ratios at which each continuity appeared when 100 molecules were randomly arranged in a 0.68:0.32 ratio of molecules 6 and 7, then randomly mutated (molecule 6 and molecule 7 were randomly swapped). This can be calculated by creating a simple Excel sheet.

The replication conditions of polymerized molecule 1 were the same as those of the previous model, with the following conditions: molecule 11 is present in the cell and there are a sufficient number of molecules 6 and 7. In this model, a setting in which a mutation occurs by the random exchange of molecules 6 and 7 with a certain probability was introduced.

### Model implementation and calculation conditions

#### Configuration of lattice cell grids

The model was programmed in JavaScript language. This model used 2D hexagonal grids in which the transition rules were simple to apply. Although square grids are generally used in 2D CA modeling, hexagonal grids were also used because they are isotropic compared with square grids. This model was implemented under the following conditions:

- Calculation field: 100 × 100 cells in hexagonal grids.
- Periodic boundary condition.

#### Conditions of calculations

The diffusion of molecules can vary according to the residual rate of each molecule, and the values in Table 1 were set as the standard case, referring to the settings in the previous Ishida’s model. A higher residual rate implied a higher percentage of molecules remaining in the lattice cell. Moreover, a smaller value resulted in a more rapid diffusion.

**Table 1:** Standard parameters and the settings used for calculations in cases with modified parameters for the standard case.

	Residual rate with diffusion process	Initial arranged number of entire computational space	Initial arranged number of central initial placement area
Molecule 1	0.0	1,000,000	
Molecule 2	0.75		100
Molecule 3	0.05		100
Molecule 4	= Molecule 2		
Molecule 5	= Molecule 4		
Molecule 6	0.0	50,000	
Molecule 7	0.0	50,000	
Molecule 8	1.0		
Molecule 9	1.0		
Molecule 10	1.0		
Molecule 11	1.0		
Molecule 12	0.0	100,000	
Molecule 13	1.0		
Molecule 14	1.0	3	
Molecule 15	1.0	9	
Molecule 19	0.0		
Molecule 20	0.4	100	1000
Molecule 25	0.2	100	500
Polymerized molecule 1	0.75	0	10
Polymerized molecule 2	0.75	0	

#### Initial conditions

As an initial condition, molecule 1 was placed throughout the lattice with 1,000,000 molecules in each cell, molecules 6 and 7 with 50,000 molecules, and molecule 12 with 100,000 molecules. Concomitantly, 100 energy resource molecules 20 and 100 energy molecules 25 were placed.

In addition, 100 molecules 2 and 3 in some cells were placed in the central area of the lattice field (Fig. 9B). In addition, 10 polymerized molecules 1, which were polymerized with ratio molecules 6 and 7 with morphological parameter w, were placed in the central region of the space, as shown in Fig. 9C.

For the determination of the standard case value of each parameter, the parameters were adjusted through several trials and errors, in reference to the values of the previous Ishida model^22^. Table 1 lists the values of each parameter for the standard case.

#### Calculation case

The effects of the major parameters on the calculation results are summarized in the Ishida model^22^. The parameters investigated in this study were the amount of energy resources E (the number of molecules 20) supplied to the lattice space at each computational step and the mutation rate of the polymerized molecule 1. In each cell, it was assumed that the following number of molecules 20 would be supplied in the next step if molecule 20 was zero.

$$ {\text{E}} = {2}00,{ 3}00,{ 4}00,{ 5}00,{ 6}00,{ 7}00,{ 8}00. $$

In addition, to simulate an environment in which the energy resources decreased over time, a setting in which energy resources E were reduced after a certain time step was also used in calculations at the same time. The number of resources was reduced to 200 after the 4000th step and to 100 after the 6000th step relative to the initial supply of resources. Furthermore, the mutation rate was calculated for 0.1, 0.2, 0.3, 0.4, and 0.5 using 0.1 as the standard.

## Data Availability

Simulation Video of Standard Parameters case (Fig. 2A) can be seen at https://youtu.be/McU-nUWXj-w (accessed on 26 April 2023). The source code of the simulation model can be downloaded from the following; https://github.com/Takeshi-Ishida/Simulation-of-the-emergence-of-cell-like-morphologies-with-evolutionary-potential (accessed on 26 April 2023).
